# Supplementary material for: HIVprotI: an integrated web based platform for prediction and design of HIV proteins inhibitors
Source: J Cheminform. 2018 Mar 9;10:12. doi: 10.1186/s13321-018-0266-y (PMC5845081; doi:10.1186/s13321-018-0266-y)
Supplement: Supplementary file 2 — Additional file 2. Source code of HIVProtI web server. [file 13321_2018_266_MOESM2_ESM.zip › HIVprotI_Source-code/hivprotI/weka-3-6-6/doc/weka/core/neighboursearch/balltrees/BallNode.html]

BallNode


---


|  |  |  |
| --- | --- | --- |
| **Overview** \| **Package** \| **Class** \| **Tree** \| **Deprecated** \| **Index** \| **Help** \| **Weka's home** | |  |
| PREV CLASS   **NEXT CLASS** | **FRAMES**    **NO FRAMES**     **All Classes** |  |
| SUMMARY: NESTED \| FIELD \| CONSTR \| METHOD | DETAIL: FIELD \| CONSTR \| METHOD |  |


---


## weka.core.neighboursearch.balltrees Class BallNode

```
java.lang.Object
  weka.core.neighboursearch.balltrees.BallNode
```

**All Implemented Interfaces:**: java.io.Serializable, RevisionHandler

---

``` public class BallNode extends java.lang.Object implements java.io.Serializable, RevisionHandler ```

Class representing a node of a BallTree.

**Version:**
:   $Revision: 1.2 $

**Author:**
:   Ashraf M. Kibriya (amk14[at-the-rate]cs[dot]waikato[dot]ac[dot]nz)

**See Also:**: Serialized Form

---

| **Field Summary** | |
| --- | --- |
| `int` | `m_End`             The end index of the portion of the master index array, which stores indices of the instances/points the node contains. |
| `BallNode` | `m_Left`             The left child of the node. |
| `int` | `m_NodeNumber`             The node number/id. |
| `int` | `m_NumInstances`             The number of instances/points in the node. |
| `BallNode` | `m_Right`             The right child of the node. |
| `int` | `m_SplitAttrib`             The attribute that splits this node (not always used). |
| `double` | `m_SplitVal`             The value of m\_SpiltAttrib that splits this node (not always used). |
| `int` | `m_Start`             The start index of the portion of the master index array, which stores the indices of the instances/points the node contains. |


**Constructor Summary**

`BallNode(int nodeNumber)`
  
          Constructor.

`BallNode(int start,
int end,
int nodeNumber)`
  
          Creates a new instance of BallNode.

`BallNode(int start,
int end,
int nodeNumber,
Instance pivot,
double radius)`
  
          Creates a new instance of BallNode.


| **Method Summary** | |
| --- | --- |
| `static Instance` | `calcCentroidPivot(int[] instList, Instances insts)`             Calculates the centroid pivot of a node. |
| `static Instance` | `calcCentroidPivot(int start, int end, int[] instList, Instances insts)`             Calculates the centroid pivot of a node. |
| `static Instance` | `calcPivot(BallNode child1, BallNode child2, Instances insts)`             Calculates the centroid pivot of a node based on its two child nodes (if merging two nodes). |
| `static double` | `calcRadius(BallNode child1, BallNode child2, Instance pivot, DistanceFunction distanceFunction)`             Calculates the radius of a node based on its two child nodes (if merging two nodes). |
| `static double` | `calcRadius(int[] instList, Instances insts, Instance pivot, DistanceFunction distanceFunction)`             Calculates the radius of node. |
| `static double` | `calcRadius(int start, int end, int[] instList, Instances insts, Instance pivot, DistanceFunction distanceFunction)`             Calculates the radius of a node. |
| `Instance` | `getPivot()`             Returns the pivot/centre of the node's ball. |
| `double` | `getRadius()`             Returns the radius of the node's ball. |
| `java.lang.String` | `getRevision()`             Returns the revision string. |
| `boolean` | `isALeaf()`             Returns true if the node is a leaf node (if both its left and right child are null). |
| `int` | `numInstances()`             Returns the number of instances in the hyper-spherical region of this node. |
| `void` | `setPivot(Instance pivot)`             Sets the pivot/centre of this nodes ball. |
| `void` | `setRadius(double radius)`             Sets the radius of the node's ball. |
| `void` | `setStartEndIndices(int start, int end)`             Sets the the start and end index of the portion of the master index array that is assigned to this node. |

**Methods inherited from class java.lang.Object**

`equals, getClass, hashCode, notify, notifyAll, toString, wait, wait, wait`

**Field Detail**

### m\_Start

```
public int m_Start
```

:   The start index of the portion of the master index array,
    which stores the indices of the instances/points the node
    contains.

---


### m\_End

```
public int m_End
```

:   The end index of the portion of the master index array,
    which stores indices of the instances/points the node
    contains.

---


### m\_NumInstances

```
public int m_NumInstances
```

:   The number of instances/points in the node.

---


### m\_NodeNumber

```
public int m_NodeNumber
```

:   The node number/id.

---


### m\_SplitAttrib

```
public int m_SplitAttrib
```

:   The attribute that splits this node (not
    always used).

---


### m\_SplitVal

```
public double m_SplitVal
```

:   The value of m\_SpiltAttrib that splits this
    node (not always used).

---


### m\_Left

```
public BallNode m_Left
```

:   The left child of the node.

---


### m\_Right

```
public BallNode m_Right
```

:   The right child of the node.


**Constructor Detail**

### BallNode

```
public BallNode(int nodeNumber)
```

:   Constructor.

    **Parameters:**: `nodeNumber` - The node's number/id.

---


### BallNode

```
public BallNode(int start,
                int end,
                int nodeNumber)
```

:   Creates a new instance of BallNode.

    **Parameters:**: `start` - The begining index of the portion of the master index array belonging to this node.: `end` - The end index of the portion of the master index array belonging to this node.: `nodeNumber` - The node's number/id.

---


### BallNode

```
public BallNode(int start,
                int end,
                int nodeNumber,
                Instance pivot,
                double radius)
```

:   Creates a new instance of BallNode.

    **Parameters:**: `start` - The begining index of the portion of the master index array belonging to this node.: `end` - The end index of the portion of the master index array belonging to this node.: `nodeNumber` - The node's number/id.: `pivot` - The pivot/centre of the node's ball.: `radius` - The radius of the node's ball.


**Method Detail**

### isALeaf

```
public boolean isALeaf()
```

:   Returns true if the node is a leaf node (if
    both its left and right child are null).

    :   **Returns:**: true if the node is a leaf node.

---


### setStartEndIndices

```
public void setStartEndIndices(int start,
                               int end)
```

:   Sets the the start and end index of the
    portion of the master index array that is
    assigned to this node.

    :   **Parameters:**: `start` - The start index of the master index array.: `end` - The end index of the master indext array.

---


### setPivot

```
public void setPivot(Instance pivot)
```

:   Sets the pivot/centre of this nodes
    ball.

    :   **Parameters:**: `pivot` - The centre/pivot.

---


### getPivot

```
public Instance getPivot()
```

:   Returns the pivot/centre of the
    node's ball.

    :   **Returns:**: The ball pivot/centre.

---


### setRadius

```
public void setRadius(double radius)
```

:   Sets the radius of the node's
    ball.

    :   **Parameters:**: `radius` - The radius of the nodes ball.

---


### getRadius

```
public double getRadius()
```

:   Returns the radius of the node's ball.

    :   **Returns:**: Radius of node's ball.

---


### numInstances

```
public int numInstances()
```

:   Returns the number of instances in the
    hyper-spherical region of this node.

    :   **Returns:**: The number of instances in the node.

---


### calcCentroidPivot

```
public static Instance calcCentroidPivot(int[] instList,
                                         Instances insts)
```

:   Calculates the centroid pivot of a node. The node is given
    in the form of an indices array that contains the
    indices of the points inside the node.

    :   **Parameters:**: `instList` - The indices array pointing to the instances in the node.: `insts` - The actual instances. The instList points to instances in this object. **Returns:**: The calculated centre/pivot of the node.

---


### calcCentroidPivot

```
public static Instance calcCentroidPivot(int start,
                                         int end,
                                         int[] instList,
                                         Instances insts)
```

:   Calculates the centroid pivot of a node. The node is given
    in the form of the portion of an indices array that
    contains the indices of the points inside the node.

    :   **Parameters:**: `start` - The start index marking the start of the portion belonging to the node.: `end` - The end index marking the end of the portion in the indices array that belongs to the node.: `instList` - The indices array pointing to the instances in the node.: `insts` - The actual instances. The instList points to instances in this object. **Returns:**: The calculated centre/pivot of the node.

---


### calcRadius

```
public static double calcRadius(int[] instList,
                                Instances insts,
                                Instance pivot,
                                DistanceFunction distanceFunction)
                         throws java.lang.Exception
```

:   Calculates the radius of node.

    :   **Parameters:**: `instList` - The indices array containing the indices of the instances inside the node.: `insts` - The actual instances object. instList points to instances in this object.: `pivot` - The centre/pivot of the node.: `distanceFunction` - The distance fuction to use to calculate the radius. **Returns:**: The radius of the node. **Throws:**: `java.lang.Exception` - If there is some problem in calculating the radius.

---


### calcRadius

```
public static double calcRadius(int start,
                                int end,
                                int[] instList,
                                Instances insts,
                                Instance pivot,
                                DistanceFunction distanceFunction)
                         throws java.lang.Exception
```

:   Calculates the radius of a node.

    :   **Parameters:**: `start` - The start index of the portion in indices array that belongs to the node.: `end` - The end index of the portion in indices array that belongs to the node.: `instList` - The indices array holding indices of instances.: `insts` - The actual instances. instList points to instances in this object.: `pivot` - The centre/pivot of the node.: `distanceFunction` - The distance function to use to calculate the radius. **Returns:**: The radius of the node. **Throws:**: `java.lang.Exception` - If there is some problem calculating the radius.

---


### calcPivot

```
public static Instance calcPivot(BallNode child1,
                                 BallNode child2,
                                 Instances insts)
                          throws java.lang.Exception
```

:   Calculates the centroid pivot of a node based on its
    two child nodes (if merging two nodes).

    :   **Parameters:**: `child1` - The first child of the node.: `child2` - The second child of the node.: `insts` - The set of instances on which the tree is (or is to be) built. **Returns:**: The centre/pivot of the node. **Throws:**: `java.lang.Exception` - If there is some problem calculating the pivot.

---


### calcRadius

```
public static double calcRadius(BallNode child1,
                                BallNode child2,
                                Instance pivot,
                                DistanceFunction distanceFunction)
                         throws java.lang.Exception
```

:   Calculates the radius of a node based on its two
    child nodes (if merging two nodes).

    :   **Parameters:**: `child1` - The first child of the node.: `child2` - The second child of the node.: `pivot` - The centre/pivot of the node.: `distanceFunction` - The distance function to use to calculate the radius **Returns:**: The radius of the node. **Throws:**: `java.lang.Exception` - If there is some problem in calculating the radius.

---


### getRevision

```
public java.lang.String getRevision()
```

:   Returns the revision string.

    :   **Specified by:**: `getRevision` in interface `RevisionHandler`
    :   **Returns:**: the revision


---


|  |  |  |
| --- | --- | --- |
| **Overview** \| **Package** \| **Class** \| **Tree** \| **Deprecated** \| **Index** \| **Help** \| **Weka's home** | |  |
| PREV CLASS   **NEXT CLASS** | **FRAMES**    **NO FRAMES**     **All Classes** |  |
| SUMMARY: NESTED \| FIELD \| CONSTR \| METHOD | DETAIL: FIELD \| CONSTR \| METHOD |  |


---
